# Face Inversion Effect on Perceived Cuteness and Pupillary Response

**DOI:** 10.3389/fpsyg.2020.558478

**Published:** 2020-09-03

**Authors:** Kana Kuraguchi, Kei Kanari

**Affiliations:** ^1^Faculty of Psychology, Otemon Gakuin University, Osaka, Japan; ^2^Department of Fundamental Engineering, Utsunomiya University, Tochigi, Japan

**Keywords:** face, cuteness, attractiveness, face inversion effect, pupillary response

## Abstract

The face inversion effect reflects the special nature of facial processing and appears not only in recognizing facial identity or expression but also in subjective evaluation, such as facial attractiveness. Previous studies have revealed that the way in which we perceive attractiveness (beauty versus cuteness) differs our perceptual behavior. Therefore, the face inversion effect on attractiveness might differ based on the viewpoint of attractiveness. In this study, we measured pupillary response when judging the cuteness of facial stimuli and focused on the mechanism of perceiving attractiveness in terms of the effect of involuntary physical reaction. We investigated whether perceived cuteness – a kind of attractiveness – was affected by face inversion and whether the face inversion effect appeared in pupillary responses. We then conducted experiments in which participants observed inverted faces and rated the subjective cuteness of the faces, and we measured the participants’ pupil size while they observed the facial stimuli. The results revealed a negative correlation between pupil changes and the perceived cuteness of inverted faces, which is consistent with the previous result of upright faces. Thus, we found that the perception of facial cuteness is little affected by face inversion, suggesting that the judgment of cuteness is processed differently from other types of attractiveness such as beauty. We also found that pupillary response is related to perceiving cuteness, which could lead to consistency in the perception of cuteness.

## Introduction

Facial attractiveness affects our decisions, not only in mate selection but also in daily life. Perceiving attractiveness can be divided into different aspects of attractiveness, such as cuteness and beauty. In particular, feeling cuteness can motivate us to take care of someone, such as babies or infants. Evoking cuteness also differs from other aspects of attractiveness in terms of facial perception. Perceiving the cuteness of a face is brought about by baby schema: a set of infantile physical features, such as large eyes, a high and protruding forehead, chubby cheeks, and a small mouth (Lorenz, 1943). Hence, cuteness has been treated and investigated primarily as a child’s attraction (Alley, 1981; Glocker et al., 2009; Sprengelmeyer et al., 2009; Lobmaier et al., 2010; Little, 2012), even though cuteness can also be judged in adult female faces (Kuraguchi and Ashida, 2015; Kuraguchi et al., 2015). In contrast, perceiving the beauty of a face is treated primarily as the index of mate selection (Rhodes, 2006) and is mainly aroused by symmetry (Grammer and Thornhill, 1994; Rhodes et al., 1998), averageness (Langlois and Roggman, 1990), and sexual dimorphism (Perrett et al., 1998; Johnston et al., 2001; Penton-Voak et al., 2001; Feinberg et al., 2005). Thus, perceiving cuteness mainly depends upon individual facial characteristics such as the size of eyes or mouth, whereas perceiving beauty mainly depends upon holistic features. This means that perceptual processing of facial cuteness differs from other forms of attractiveness represented by beauty and that specific situations that disrupt facial processing – for instance, face inversion – affect perceiving cuteness differently. Cuteness differs qualitatively from other aspects of attractiveness represented by beauty, which is also found in the fact that our perception of cuteness is harder to judge in the peripheral vision than in the central vision, although beauty is equally possible in both (Kuraguchi and Ashida, 2015). In other words, the perception of cuteness involves perceptual changes, such as narrowing of the range of attention (Nittono et al., 2012; Kuraguchi and Ashida, 2015), which directs attention toward the object in front of the perceiver and may lead to an expression of the caretaking motivation. The perceptual characteristics of feeling cuteness may appear in specific situations, such as when observing someone in the peripheral vision or in recognizing upside-down faces. We therefore conducted an experiment to rate the cuteness of inverted faces, which is difficult in facial processing, in order to discover the perceptual characteristics of feeling cuteness.

Face inversion disrupts our facial processing, and we focused on the face inversion effect to investigate how facial cuteness is perceived. Facial recognition depends on holistic processing – perceiving facial configuration as a gestalt – which can be divided into two types of processing: *first-order relational processing* of the arrangement of features (e.g., two eyes above a nose) and *second-order relational processing* of the special distances among internal features (reviewed in Maurer et al., 2002). Yin (1969) reported that face inversion disrupts the recognition of facial identity. Later studies have suggested that this face inversion effect is caused by the disruption of holistic or second-order relational processing through experiments with face images (Young et al., 1987; Hole, 1994; Freire et al., 2000; Barton et al., 2003), although first-order relationship may be inhibited by the inversion of stimuli that are visible as faces, such as a moony face, because it hardly perceives them as faces when they are turned upside down (Maurer et al., 2002). Furthermore, it is suggested that the face inversion effect is a consequence of disruption of the perceptual process because the perceptual field is constricted for inverted faces (Rossion, 2008, 2009). Face inversion might affect not only facial identity but also various aspects of facial recognition. For example, the recognition of gender is disrupted by face inversion, which is considered to be caused by a disruption in information about facial relational configuration (Bruce et al., 1993). Facial size estimation also differs between upright and inverted faces (Araragi et al., 2012; Thompson and Wilson, 2012).

Face inversion is suggested to affect perceived attractiveness represented by beauty. Bäuml (1994) conducted an experiment in which participants had to select the most attractive face between two or among three male faces, and found that the preference was closer to indifferent choice (i.e., chance level) when faces were inverted than when they were upright. Abbas and Duchaine (2008) investigated the composite face effect on judgments of facial attractiveness when aligned and misaligned halves of faces were presented in upright and inverted viewings, and they suggest that facial attractiveness is judged through holistic processing. Additionally, face inversion increased rating scores of the attractiveness of faces, except for originally highly attractive faces in upright viewing positions (Leder et al., 2017). This may be due to the fact that the absence of unattractive features is taken into account in the assessment of facial attractiveness. Similarly, inverted unattractive faces are rated more highly regardless of presentation time, while inverted attractive faces are rated lower (Stróżak and Zielińska, 2019). The inversion effect on perceived attractiveness might be related to difficulty in recognizing the factors of attractiveness when faces are inverted. For example, the degree of symmetry affects facial attractiveness, but a greater preference for facial symmetry has been shown to arise when faces were upright than when they were inverted (Little and Jones, 2003). A happy facial expression, such as a smile, enhances perceived attractiveness (Golle et al., 2014; Ueda et al., 2016), but recognition of facial expressions is also disrupted by face inversion (Sugase-Miyamoto et al., 2014).

According to the findings mentioned above, face inversion makes it difficult to accurately evaluate attractiveness based on the disruption of second-order relational processing or holistic processing, or because of the constriction of perceptual field, which causes an inversion effect on perceived attractiveness represented by beauty. However, cuteness may differ from beauty in terms of perceptual processing and may not be affected by face inversion. Thus, perceiving cuteness may be processed sufficiently by first-relational processing alone because cuteness is evoked by individual facial characteristics. Moreover, Matsuyoshi et al. (2015) revealed that the face inversion effect is aroused by not suppressing the brain area that relates to object processing, which means that inverted faces are ambiguous in terms of whether they are faces or objects, although face processing is robustly done in inverted faces (Prete et al., 2015). The inhibition of object processing in the upright viewing position makes facial processing smoother and may adjust facial beauty finely because facial beauty requires perceiving holistic features, such as symmetry, which are disrupted by face inversion. On the other hand, perceiving the cuteness of inverted faces might not be largely affected by failing to suppress object processing because the facial features of baby schema, such as large eyes and small mouth, are piecemeal. Therefore, the face inversion effect may not appear in the rating scores of facial cuteness.

Perceiving cuteness alters our pupillary response depending on the evaluation targets. Kuraguchi and Kanari (submitted) revealed a negative correlation between pupillary response and the perceived cuteness of female faces in upright viewing positions but a positive correlation between pupillary response and the perceived cuteness of images other than human faces, such as animals and foods. Note that the effects of image luminance on pupillary response were controlled because this experiment was conducted after the mean luminance of the images was adjusted, and the effects of the standard deviation of luminance were also excluded by partial correlation. If perceiving the cuteness of inverted faces is based on object processing, we will find a positive correlation between pupillary response and the cuteness ratings of inverted faces. In contrast, if perceiving the cuteness of inverted faces is based on facial processing, we will find a negative correlation between them. We thus investigated whether pupillary response correlated negatively to cuteness ratings of inverted faces and whether the perception of the cuteness of inverted faces processed them as faces but not objects. Pupillary response is an involuntary reaction to stimuli, and thus, a reliable index of consistency between upright and inverted viewing positions.

In this study, we investigated whether perceived cuteness was affected by face inversion and whether the face inversion effect appeared in pupillary responses. Therefore, we conducted an experiment in which participants observed inverted faces and rated the subjective cuteness of the faces, and we measured the participants’ pupil size while they observed the stimuli and calculated the percentage increase in pupillary size based on the size before the stimulus presentation. Previous studies suggest that females are more sensitive to the perception of cuteness (Sprengelmeyer et al., 2009; Lobmaier et al., 2010, 2015; Hahn et al., 2013; Lehmann et al., 2013; Nittono, 2016). Therefore, female participants were sought for this study.

## Materials and Methods

### Participants

The participants who observed inverted faces included 14 Japanese females (age range: 18–25 years, mean age: 20.78, all right-handers), who were unaware of the purpose of the experiment. All reported normal or corrected-to-normal vision. We obtained written informed consents from all participants before beginning the experiment and paid a reward according to the standard of Otemon Gakuin University. This study was approved by the Ethics Committee of Otemon Gakuin University (Approval Number: 2019-15) and was conducted in accordance with the Code of Ethics of the World Medical Association (Declaration of Helsinki).

The data of the participants who observed upright faces were analyzed using the corresponding data (14 Japanese females, age range: 18–25 years, mean age: 20.71) of Kuraguchi and Kanari (submitted). Eleven females in this study also participated in the experiment of Kuraguchi and Kanari (submitted).

### Stimuli

The stimuli consisted of the 2D facial images of 20 Japanese females (18–25 years, undergraduate and graduate students) from a frontal view and with a neutral expression. The stimulus subtended 14.0 × 14.0 deg (512 × 512 pixels). All images were presented in an inverted view. Because this study was conducted on Japanese women, we collected their facial images. However, as very few databases of Japanese faces are available to the public, we did not use the available databases but recruited and photographed subjects ourselves. Before the images were used in the experiment as stimuli, we tested whether the photographed faces had neutral expressions (i.e., did not appear to have any particular expression) using a different set of participants from the study participants. All images were included in the stimuli (25 faces) used in Kuraguchi and Kanari (submitted). We used a minimum of facial images in order to reduce the participants’ burden when observing images.

All face images were converted to grayscale using the “rgb2gray” function (NTSC standard) in MATLAB (The MathWorks) and normalized using the following formula:

I=norm,i(I-iI)min/(I-maxI)min

where *I*_*norm,i*_ is the normalized pixel intensity, *I*_*i*_ is the original intensity, *I*_*max*_ is the maximum intensity, and *I*_*min*_ is the minimum intensity. Then, the mean luminance of the normalized image was manipulated with a gamma correction using the “imadjust” function in MATLAB. The mean luminance of all images was 20.15 cd/m^2^ (*SD* = 0.17, *Min* = 20.00, *Max* = 20.49). The luminance of the display (SONY GDM F500R) was measured using a luminance meter (LS-110, KONICA MINOLTA Holding Inc.), while a grayscale circle with a diameter of 2° was presented in increments of 10 Red–Green–Blue (RGB) values from 0–255 (with a final value of 255 following 250). The luminance of the image was estimated based on this table.

### Procedure

All trials began with instructions that told participants to rate the subjective cuteness of the presented stimuli. Following a participant’s button press, a mask stimulus consisting of scrambled dots (mean luminance 20.0 cd/m^2^) and a fixation point in the center of the screen was presented for 3 s. Then, a test stimulus (a face image in an inverted view) was presented at the center of the display for 4 s. Stimulus presentation time (4 s) was determined from the reaction time of the pupil light reflex (Ellis, 1981). Participants rated the cuteness of the face on a 7-point scale (1 = not cute; 7 = very cute) by pressing an assigned key. Participants responded by using a numeric keypad to their right side, but they were not instructed which hand to use. After the key press, the participants could not press the button again to launch the following trial for 3 s. Each participant performed three blocks, which resulted in 60 trials in total. In each block, 20 trials were presented in a random order. This procedure was done in the same way as Kuraguchi and Kanari (submitted), except that the test stimuli were presented upside down.

### Apparatus

The stimulus was presented on a 21 in. Cathode Ray Tube (SONY GDM F500R) at a viewing distance of 57 cm. The refresh rate of the display was 60 Hz, and the resolution was 1280 × 960 pixels (35.1 × 28.9°, 36 × 29.4 cm). The display was specifically designed for the precise manipulation of luminance. The participants sat in a dark room and observed the stimuli with their heads fixed by a chin rest. Stimuli were generated using Psychophysics Toolbox extensions (Brainard, 1997; Pelli, 1997) for MATLAB (The MathWorks) and presented using a MacBook Pro (Apple). This apparatus and settings were the same as Kuraguchi and Kanari (submitted).

### Pupillometry

The pupil size of the right eye was measured using an infrared sensitive camera (EyeLink 1000 Desktop Mount, SR Research) with a sampling rate of 1000 Hz. The eye tracker was calibrated before the experimental phase using a 9-point calibration grid. Periods of blinks were detected using the EyeLink’s standard algorithms with the default setting. Data from 150 milliseconds (ms) before the onset of blinks to 150 ms after the offset of blinks were excluded from the analysis. In addition, trials were excluded if more than 35% of the data within that trial was missing. Missing data were interpolated with a cubic spline fit. The average pupil size of the interval from -100 ms to 0 ms to the test stimulus was used as a baseline. Data obtained during the test stimulus presentation (4 s) in each trial was normalized by calculating the percentage increase of the pupil size when compared to the baseline:

[X=norm(X-databaseline)/baseline]

For each participant and stimulus, pupil data were averaged across trials. The mean change rate of pupil size during the interval 0–4 s relative to exposure to the stimulus was defined as the mean pupil change. The processing mentioned here was the same as Kuraguchi and Kanari (submitted).

## Results

### Correlation Between Mean Pupil Change and Cuteness Rating

**FIGURE 1:**
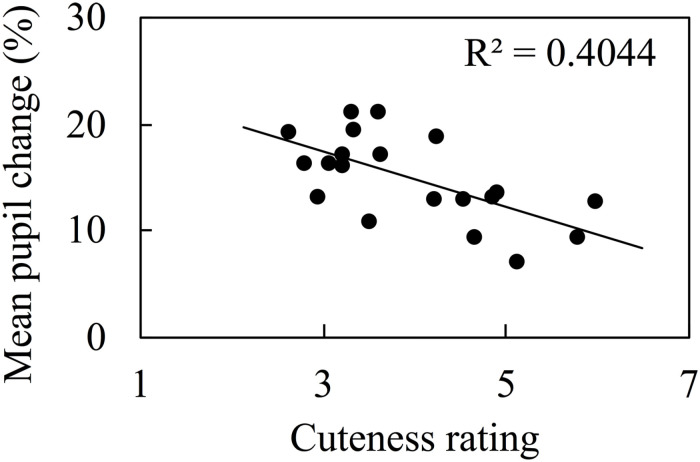
Correlation between mean pupil change and cuteness rating for inverted faces.

We used Pearson’s correlation coefficient to validate the correlation between cuteness ratings and mean pupil change in inverted face images, and normality was confirmed. This result is detailed in Figure 1. The ordinate presents the mean percentage change in pupil size from the baseline to the test stimulus (4 s). The abscissa presents the cuteness rating for the test stimulus. We calculated the correlation coefficients by Bayesian estimation method using open-source software JASP (JASP Team, 2020). The Bayes factor (BF_10_) quantifies the intensity of the evidence that the data provide for H_1_ versus H_0_ (Wagenmakers et al., 2018a,b), and we used the classification scheme adopted by JASP (1/30–1/10: strong evidence for H_0_, 1/10–1/3: moderate evidence for H_0_, 1/3–1: anecdotal evidence for H_0_, 1: no evidence, 1–3: anecdotal evidence for H_1_, 3–10: moderate evidence for H_1_, 10–30: strong evidence for H_1_, 30–100: very strong evidence for H_1_, > 100: extreme evidence for H_1_). A strong correlation between the cuteness of the image and the mean pupil change was found [*r* = -0.636, BF_10_ = 19.094, 95% credible interval (-0.823, -0.234)].

**FIGURE 2:**
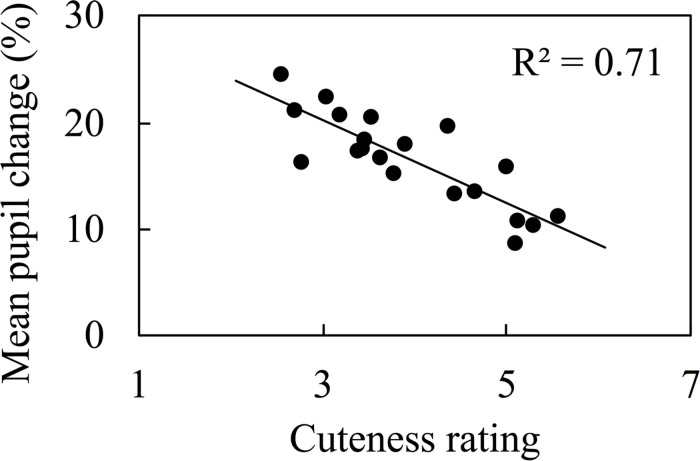
The correlation between mean pupil change and cuteness rating for upright faces. The data of upright faces were adopted from Kuraguchi and Kanari (submitted).

For reference, we picked up the corresponding data for upright face images from the results of Kuraguchi and Kanari (submitted) and recalculated the correlation coefficient between the rated cuteness of the upright faces and the mean pupil change, which is shown in Figure 2. The correlation between the cuteness of the images and the mean pupil change was found [*r* = -0.843, BF_10_ = 6537.853, 95% credible interval (-0.931, -0.588)]. A test (Fisher’s-r-to-z) to assess the difference between correlations in upright and inverted viewings showed no significant difference (z = -1.40, *p* = 0.164).

**FIGURE 3:**
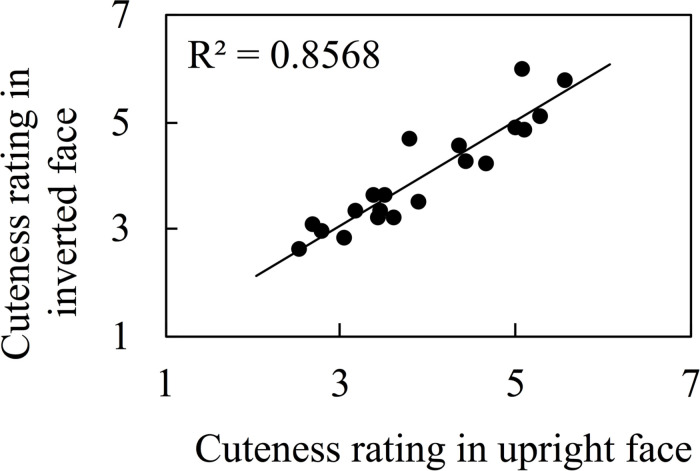
The correlation between cuteness ratings when faces were upright and inverted. The data of upright faces were adopted from Kuraguchi and Kanari (submitted).

**FIGURE 4:**
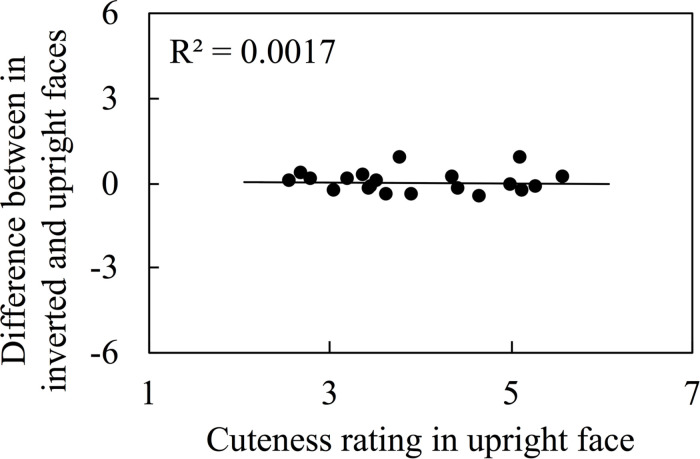
The difference of cuteness rating between in inverted and upright viewings. The data of upright faces were adopted from Kuraguchi and Kanari (submitted).

We also investigated the correlation between ratings of cuteness when presented upright from Kuraguchi and Kanari (submitted) and while upside down in this study, which is shown in Figure 3, and this revealed a positive correlation [*r* = 0.926, Log(BF_10_) = 14.472, 95% credible interval (0.780, 0.969)]. Previous studies report that face inversion decreases or increases attractiveness ratings (Leder et al., 2017; Stróżak and Zielińska, 2019). Therefore, we examined whether face inversion increases or decreases the rating scores. We plotted the difference between upright and inverted cuteness ratings on an axis of the ordinate in Figure 4. A Bayesian one-sample *t*-test revealed that the difference between ratings was not different from zero [BF_10_ = 0.252, 95% credible interval (-0.140, 0.212)].

### Temporal Changes in Pupillary Response

**FIGURE 5:**
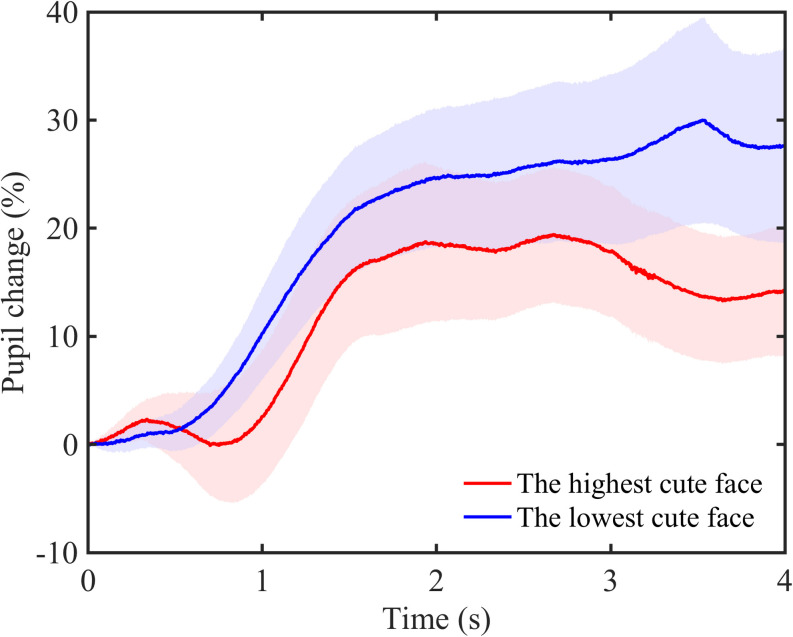
Time courses of pupillary changes during the observation of high- and low-rated faces. The red line shows the data for the highest-rated faces and blue line for the lowest-rated faces. Each colored area represents a 95% confidence interval.

We also examined temporal changes in pupillary response in inverted face image observation. We showed temporal changes in pupillary response to the highest- and lowest-rated faces by each participant and revealed that the degree of pupil dilation in low-rated faces consistently exceeded high-rated faces (Figure 5).

### Effects of Eye Size on the Relationship Between Mean Pupil Change and Cuteness Rating

The participants may have used the size of eyes as a cue to judge the cuteness of the face. To investigate this point, we examined whether the size of eyes correlated with cuteness rating. The area of both eyes as a percentage of the total face was calculated, including the sclera, pupil, and iris, and the result was used as a measure of eye size. We found that eye size correlated with cuteness rating [*r* = 0.744, BF_10_ = 197.948, 95% credible interval (0.402, 0.881)]. Because the eyes may be relatively more luminous than other parts of the face, the relatively higher luminance of the eye area in cute faces may influence pupillary response while judging cuteness in relation to the size of eyes. We then calculated the partial correlation between cuteness rating and pupil change when controlling for the proportion of both eye areas and found a significant partial correlation (*r* = -0.509, *p* = 0.025). Note that we report the *p*-values for this partial correlation because it is not possible to calculate BF for partial correlations in JASP.

## Discussion

We investigated whether perceived cuteness was affected by face inversion and whether pupillary responses related to perceiving the cuteness of faces when presented in inverted viewing positions. The result revealed the consistency of rating cuteness, regardless of face orientation, because the ratings of cuteness were correlated between upright and inverted viewing positions. We also found a negative correlation between rated cuteness and pupillary response when observing inverted faces in the same way as upright faces. These results might indicate that perceiving cuteness is little affected by face inversion, suggesting that the judgment of cuteness is processed differently from other types of attractiveness, such as beauty. In other words, the perception of facial cuteness may be primarily related to first-order processing rather than second-order processing. It may also be unaffected by the narrowing of the perceptual field.

In this study, we investigated whether the face inversion effect appeared in the rating of cuteness in the same way as beauty or attractiveness. In previous findings, facial attractiveness could be assessed through face inversion but when using different criteria from upright viewing. Bäuml (1994) found that the preference between attractive and unattractive faces was more reliable when faces were upright than when faces were inverted. Leder et al. (2017) found that face inversion increased rated attractiveness, especially for originally less attractive faces, which suggests that the absence of unattractive characteristics brought about through face inversion heightened rated attractiveness. Stróżak and Zielińska (2019) found that inverted unattractive faces are rated more highly, but inverted attractive faces are rated lower. In other words, these studies show that the accuracy of attractiveness evaluation is reduced when the face is presented as inverted, although the attractiveness evaluation is possible. In the evaluation of attractiveness, the part that provided variation in attractiveness ratings, especially in relation to the perception of unattractiveness, was considered inhibited by face inversion, suggesting that the subtractive method of assessing beauty is undermined by the inversion. The assessment of beauty or attractiveness is influenced by holistic facial features such as symmetry. Therefore, if face perception is possible, the evaluation of attractiveness may be done automatically to a certain extent, but the fine tuning of attractiveness may be based on second-order relational information of the face as well as the judgment of facial expressions. The results in this study revealed the consistency of cuteness ratings between upright and inverted viewings but not a difference in rating scores. Therefore, perceiving cuteness may be mainly judged by first-order relational processing, which is different from perceiving beauty or attractiveness, which is adjusted through second-order processing. This suggestion may be supported by the difference in facial features related to each evaluation. For example, cuteness is evoked from baby schema, such as large eyes and a small mouth, whereas beauty or attractiveness is related to facial symmetry or averageness. These differences in the evaluation of facial features created differences in the effect of face inversion on ratings. It is also possible that cuteness, unlike beauty, does not require subtractive evaluation. In other words, cuteness may be treated as evaluation that does not require the fine adjustments that beauty does. This may reflect a functional difference with beauty in that perceiving cuteness simply acts as a trigger to attract nurturing and approaching behaviors.

For pupillary responses, we found a negative correlation between pupil changes and the rating of inverted faces, which is consistent with the result for upright faces. Therefore, we suggest that perceiving the cuteness of faces is related to pupillary responses, regardless of face orientation, and the negative correlation between pupillary responses and subjective cuteness is a specific trait for facial processing. When temporal changes in pupil responses were examined, the degree of pupil enlargement for low-rated faces consistently exceeded that for high-rated faces. The consistency of pupil responses during cuteness ratings suggests that involuntary physical responses may mediate the consistency of subjective ratings, which may appear regardless of the orientation of face presentation. This may indicate that cute faces are processed more efficiently. For example, the pupils are wider in an individual performing tasks with a higher cognitive load (Porter et al., 2007; Van Der Meer et al., 2010). It is suggested that pupils are narrower when effective processing is performed in facial recognition (Goldinger et al., 2009; Hills, 2018). Hence, in facial cuteness perception, because cute faces can be judged more efficiently and easily, pupils may be less dilated. Additionally, the consistency of pupil responses in this study suggests that the disruption of perception by face inversions could have little effect on the perception of facial cuteness. Face inversion effects are also suggested to evoke as a consequence of disruption of perceptual process because the perceptual field is constricted for inverted faces (Rossion, 2008, 2009). However, we found little effect of such narrowing of the perceptual field on cuteness ratings. This may involve the fact that the perception of cuteness has a narrowing effect on attention (Nittono et al., 2012; Kuraguchi and Ashida, 2015).

Facial cuteness is defined by the baby schema. Large eyes in relation to the whole face are a characteristic of the baby schema (Lorenz, 1943). This suggests the possibility that the perception of cuteness is based on baby schema characteristics, even in inverted faces. However, the correlation with pupillary responses could not be explained by the size of eyes in the images. Therefore, we can exclude the possibility that pupil response and cuteness rating were pseudo-correlated as a result of the participants’ focus on the eye area only, suggesting that the degree of cuteness, but not the eye size of the image, was related to pupil response, even if participants did focus on the eye size while assigning ratings.

According to a previous finding (Matsuyoshi et al., 2015), the face inversion effect is aroused by not suppressing object processing. In this study, facial cuteness could be evaluated even when faces were upside down, and pupillary responses when judging facial cuteness did not differ between face orientations, which means that perceiving cuteness might not be affected by failing to suppress object processing. As a possible reason, perceiving cuteness might not need highly accurate processing of faces. Perceiving cuteness or baby schema features may be based not only on facial processing but also other mechanisms such as object recognition. Perceiving cuteness therefore may be robust enough not to fail when faces are inverted. This point should be examined in further research.

### Limitations

In this study, pupillary changes were measured before the evaluation key was pressed, and the pupillary response was not measured after the response (after pressing the evaluation key). However, the pupils were reported to be constricted after answering the question based on a single graphical input because the cognitive load disappears (Mitra et al., 2017). Although this study imposed a cuteness rating, and the quality of cognitive load was different from that of the calculations, we cannot exclude the possibility that pupils may change before and after a response. Therefore, a change in pupillary response after the evaluation judgment should be considered in the future.

We have adjusted the average luminance of the entire face, but it is not possible to adjust the luminance between the internal features of the face. Hence, when attention differs greatly from one image to the next, it is also necessary to consider the effects of the luminance of the parts. However, it is reported that the luminance of the observer’s gaze position have little effect on the higher-order processing (Naber and Nakayama, 2013). Because the perception of cuteness is a social emotion, the cuteness rating in this study is considered a higher-order process (Buckley, 2016), and the effects of the difference of luminance between facial parts were considered negligible. In addition, it is reported that the gaze pattern to the face is randomized when the face image is inverted (Barton et al., 2006). Therefore, it can be assumed that the gaze patterns for inverted faces in this study were not confined to a single location but rather to local and global scans that occurred randomly. In other words, it can be inferred that the effects of the luminance of individual parts were slight. Indeed, this idea is supported by the fact that eye size did not explain the relationship between pupillary response and rating cuteness in the results. Note, however, that since individual differences in eye movements are known to exist (Perlman et al., 2009; Peterson and Eckstein, 2013), individual differences in the relationship between pupillary response and evaluation may also exist. These points should be further examined, including interactions with the content of the ratings.

In this study, only female facial images were used as stimuli. This is because it is more common to judge female facial images as *kawaii* (cute) in Japan, but it is not common to do so for male facial images. In Japan, *kawaii* is used as an index of female gender identity (Burdelski and Mitsuhashi, 2010), which leads to a sense of discomfort in judging male adult faces under the same concept. The perception of cuteness may differ depending on the category of the subject of the evaluation because an experiment with Caucasian faces showed that female adult faces were rated cuter than male adult faces (Little, 2012). Further research should consider devising ways to investigate response to male facial images.

## Conclusion

This study found consistent ratings of cuteness in inverted faces, and this consistency was also found in pupillary response. This suggests that judgments of cuteness are less likely to be disturbed by face inversion. Therefore, it can be pointed out that cuteness was judged more roughly than beauty, and judging cuteness does not make much use of the second-order relational processing and holistic processing, which are inhibited by face inversion. It is also suggested that cuteness perception is hardly affected by constriction of the perceptual field with inversion or by failing to suppress object processing. This indicates that cuteness is evaluated by a different perceptual mechanism than that of beauty, suggesting that perceiving cuteness plays a different role from other types of attractiveness.

## Data Availability Statement

The raw data supporting the conclusions of this article will be made available by the authors, without undue reservation, to any qualified researcher.

## Ethics Statement

The studies involving human participants were reviewed and approved by the Ethics Committee of Otemon Gakuin University (Approval Number: 2019-15). The patients/participants provided their written informed consent to participate in this study.

## Author Contributions

KKu and KKa designed the study and analyzed the data. KKa performed the experiment and collected all the data. KKu drafted the manuscript. Both authors discussed the results and contributed to the final manuscript.

## Conflict of Interest

The authors declare that the research was conducted in the absence of any commercial or financial relationships that could be construed as a potential conflict of interest.
